# 0593. Reelin mediates the human neutrophil peptide-induced endothelial dysfunction and platelet aggregation

**DOI:** 10.1186/2197-425X-2-S1-P37

**Published:** 2014-09-26

**Authors:** AAL Luo, B Han, K Quinn, E Tullis, A Reheman, H Ni, A Slutsky, H Zhang

**Affiliations:** St. Michael's Hospital, Anaesthesia, Toronto, Canada; University of Toronto, Toronto, Canada; St. Michael's Hospital, Toronto, Canada

## Introduction

Atherosclerosis is an inflammatory disease with fundamental primitive events including endothelial dysfunction and platelet aggregation [1]. Human neutrophil peptides (HNP) are the most abundant cationic proteins in neutrophils and are released into the extracellular milieu upon activation [2]. HNP have previously been detected in atherosclerotic lesions, and are in high concentrations in blood of patients with acute coronary syndrome [2]. We have previously demonstrated that HNP can induce foam cell formation, platelet aggregation and leukocyte recruitment through the LRP8 signaling pathway [3], but there is no direct interaction between HNP and LRP8.

## Objectives

We examined if HNP-induced endothelial dysfunction and platelet aggregation through the LRP8-signaling pathway is mediated by the LRP ligand reelin.

## Methods

Human coronary artery endothelial cells (HCAEC) were stimulated with HNP or recombinant reelin (rRLN) to assess reelin protein expression and release, and endothelial and inducible nitric oxide synthase (eNOS and iNOS, respectively) protein expression, respectively. Human platelet-rich plasma (PRP) was primed with ADP, followed by stimulation with vehicle control, or HNP in the presence or absence of rRLN. Platelet activation and aggregation were determined by flow cytometry and Chronolog Aggregometer, respectively. The specificity of reelin-induced responses in HCAEC and PRP were confirmed by using a reelin neutralizing antibody (CR50).

## Results

HNP stimulation of HCAEC resulted in increased reelin protein expression and release. rRLN-stimulated HCAEC increased iNOS and decreased eNOS protein expression, and induced nitrotyrosine production. rRLN-treated PRP was able to produce a wave of platelet activation and aggregation that was not observed in PRP treated with ADP alone. The endothelial dysfunction and platelet aggregation responses mediated by reelin were reverted upon treatment of a reelin neutralizing antibody.

**Figure 1:**
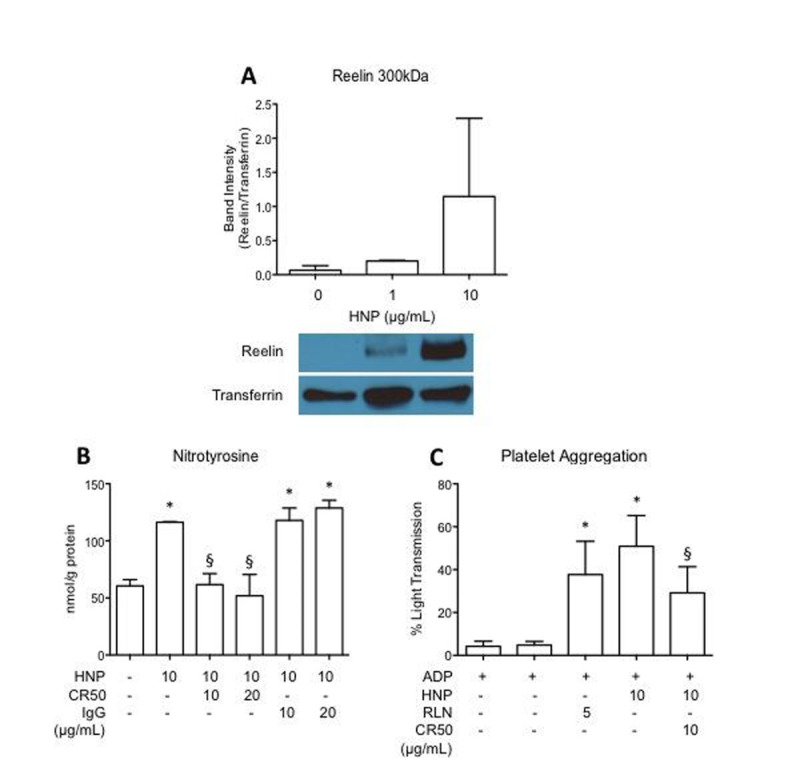
**The role of reelin in HNP-induced nitrotyrosine production and platelet aggregation.** **A.** HNP indues reelin (RLN) protein release in HCAEC. Released reelin was determined by western blot from cell culture medium. **B.** Reelin neutralizing antibody (CR50) reduced HNP-induced nitrotyrosine production. **C**. CR50 attenuates HNP-stimulated platelet aggregation in ADP-primed human platelet rich plasma. *p<0.05 *vs* control; §p<0.05 *vs* HNP 10 μg/mL.

## Conclusions

HNP-induced endothelial dysfunction, platelet activation and aggregation were mediated by the LRP8-ligand, reelin. Blocking reelin maybe a potential therapeutic target in atherosclerosis.
